# Estimation of Anonymous Email Network Characteristics through Statistical Disclosure Attacks

**DOI:** 10.3390/s16111832

**Published:** 2016-11-01

**Authors:** Javier Portela, Luis Javier García Villalba, Alejandra Guadalupe Silva Trujillo, Ana Lucila Sandoval Orozco, Tai-Hoon Kim

**Affiliations:** 1 Group of Analysis, Security and Systems (GASS), Department of Software Engineering and Artificial Intelligence (DISIA), Faculty of Information Technology and Computer Science, Office 431, Universidad Complutense de Madrid (UCM), Calle Profesor José García Santesmases, 9, Ciudad Universitaria, Madrid 28040, Spain; jportela@estad.ucm.es (J.P.); asilva@fdi.ucm.es (A.G.S.T.); asandoval@fdi.ucm.es (A.L.S.O.); 2 Department of Convergence Security, Sungshin Women’s University, 249-1 Dongseon-dong 3-ga, Seoul 136-742, Korea; taihoonn@daum.net

**Keywords:** anonymity, email network, graph theory, privacy, social network analysis, small-world-ness, statistical disclosure attack

## Abstract

Social network analysis aims to obtain relational data from social systems to identify leaders, roles, and communities in order to model profiles or predict a specific behavior in users’ network. Preserving anonymity in social networks is a subject of major concern. Anonymity can be compromised by disclosing senders’ or receivers’ identity, message content, or sender-receiver relationships. Under strongly incomplete information, a statistical disclosure attack is used to estimate the network and node characteristics such as centrality and clustering measures, degree distribution, and small-world-ness. A database of email networks in 29 university faculties is used to study the method. A research on the small-world-ness and Power law characteristics of these email networks is also developed, helping to understand the behavior of small email networks.

## 1. Introduction and Related Work

### 1.1. SNA and Email Networks

Social network analysis (SNA) has received growing attention on different areas. SNA aims to obtain relational data from social systems to identify leaders, roles, and communities in order to model profiles or predict a specific behavior in users’ network. SNA is built-in with nodes (individuals or organizations) within the network and ties representing relationships among the nodes. Social relationships may be in the form of real world offline social networks (like friendship, communications, transactions, etc.) or it may be online social networks (like Facebook, Twitter, etc.). 

SNA has been applied in Information Science [1], Financial Crimes [2], Political Science [3], Sociological studies [4], Biology [5], Economics [6], and Intelligence Analysis [7].

With regard to the properties and structures on the network, SNA has been applied to study the structure of Internet graph topologies [8], telecommunication graphs [9], emails and social networks [10,11,12]. 

Analysis of incomplete social networks where some nodes or edges are missing and only a sample of them is available is a field studied in [13,14]. A particular issue in this field is link prediction where, given some information about present nodes and links, the challenge is to fill or predict missing links in the network. See [15,16] for two surveys on link prediction. The main contribution of this article is focused on email networks, as a subdomain of social networks. Studies of email networks cover spam analysis [14,17,18], virality studies [12], community characteristics [10], structure and properties analysis [8,12], and studies on the temporal evolution of email data [19].

### 1.2. Privacy in Communication Networks

Social networks, and in particular email networks, need to be anonymized in order to preserve privacy of actors. Anonymity can be compromised by disclosing senders’ or receivers’ identity, message content, or sender-receiver relationships. Anonymity systems provide mechanisms to enhance user privacy and to protect computer systems. Research in this area focuses on developing, analyzing, and executing anonymous communication networks attacks and defenses.

Mixes [20] are considered the base for building high latency anonymous communication systems. A mix network aims to hide the correspondences between the items in its input and those in its output, changing the incoming packets’ appearance through cryptographic operations. Babel [21], Mixmaster [22], Mixminion [23], and Onion routing [24] are other anonymous communication designs. 

The attacks against mix systems aim to reduce the anonymity by linking senders with the messages that they send, receivers with the messages that they receive or linking senders with receivers. There are intersection attacks based on traffic analysis. One of the first models to appear was called the Disclosure Attack [25]. In this first version the disclosure of links between users of a network was limited to relationships of one specific user with the rest of users in the domain, under an optimization approach. 

Using the restrictive assumptions set in [25], the Statistical Disclosure Attack (SDA) was presented in [26]. In this attack, the information retrieved by the attacker is obtained through several rounds of communications, and statistical methods are employed to infer links between users. Other forms and derivations of the Statistical Disclosure Attack are presented in works [27,28,29,30]. In [31,32] a new statistical disclosure attack is presented that overcomes the restrictions usually considered in other methods and can be used under very general assumptions.

This article addresses the research problem of obtaining global and node characteristics of an anonymized email network through the application of a statistical disclosure attack. The techniques developed in [31,32] are used to infer email network characteristics under strongly incomplete information. It is shown that the attack can lead to obtain moderately accurate estimators for measures such as average degree, power law coefficient, betweenness coefficient or small-world-ness coefficient. These estimations can be used to study temporal evolution of the network, identifying substantial changes over time, compare different networks, or identify important users or clusters of users. The database used, that comprises many networks, allows study of the scope and limitations of the method under a broad statistical perspective, observing patterns, error rates, and possible biases in the estimations. This is a novelty, since there is not previous statistical disclosure attack (SDA) research on estimating global network characteristics or node based measures; generally SDA existing research focuses on a limited number of users and establishes very restricted hypotheses about a priori knowledge of the network or users behavior.

Most often analysis of email networks are restricted to only one email network; studies that treat many networks usually consider besides email data, social network data, patents data, etc. When characteristics of email networks such as small-world-ness coefficient, density or degree distribution parameter are studied for only one network, it is difficult to obtain information about the real range and variability of these parameter values. In this context, statistical variability between different small email networks can only be estimated from an ‘article to article’ perspective. Parameter variability can then be due to the different sources. There could be also a bias due to it being easier to publish papers that agree with the range of parameters in previous literature. In this case, variability could be underestimated. Also, with only one network, relationships between parameters cannot be studied.

In this article, there is not one, but a range of small email networks, each one collected at a different faculty in the same university. It allows not only to study if the parameter behavior agree with previous research but also to study the range and variability of parameters, as well as relationships between them. This is an important novelty with respect to previous research on email network data.

The rest of the article is structured as follows: Section 2 presents structural properties and measures of social networks, focusing on the particular case of email networks and their scale free or small-world characteristics. A new email database of 29 email local university networks is introduced to explore the consistency of theory. Section 3 presents the problem of retrieving network characteristics under the situation of strongly incomplete information. A statistical disclosure attack method is used to estimate the network and node measures. The method is applied to the university email database in order to study its performance. Section 4 provides conclusions and future work.

## 2. Social Networks and Email Network Properties: Email Database

The most significant structural properties of social networks are first introduced. Social Networks can be directed or undirected, weighted or unweighted. An email network can be set as directed and weighted; for simplicity, some characteristics can be computed as for an undirected and/or unweighted network. Measures of interest of a network can be divided into node centered measures and global measures.

At node level the most important measures are:

- **Degree**: The centrality degree of a node is the number of users or nodes that are directly related to it. Two nodes of a graph are adjacent or neighbors if there is a branch that connects them. In the case of directed graphs, there are two types of degrees: The input degree of a node is the number of arcs that end in it. The output degree of a node is the number of arcs that originate from it.
- **Betweenness centrality measure**: It is equal to the number of shortest paths from all vertices to all others that pass through that node. The computation of shortest paths in a network algorithms such as Floyd-Warshall or Johnson’s. A node with high betweenness centrality has a large influence on the transfer of items-messages through the network.
- **Clustering coefficient**: It is a metric that measures the extent to which the neighbors of a node are also interconnected. Here the Watts and Strogatz ([33]) local clustering coefficient is used. The clustering coefficient of a node v is defined as
  (1) $$Cv=\frac{2Ev}{kv\left(kv-1\right)}$$
  where, kv denotes the number of neighbors of v, $\frac{kv\left(kv-1\right)}{2}$ the maximum number of edges that can exist between the neighbors, and Ev the number of the edges that actually exist.
- **Closeness**: The degree of closeness is the ability of a node to reach all others in the network. A node is important if it is close to all others. The sum of the shortest path distance from the node ni to all the others is computed. The inverse of this sum is the closeness coefficient of node $n_{i}$:
  (2) $$C_{c}\left(n_{i}\right)={\left[\overset{g}{\underset{j=1}{\sum }}d\left(n_{i},n_{j}\right)\right]}^{-1}$$
  where $d\left(n_{i},n_{j}\right)$ is the shortest path distance between nodes *i* and *j*. While betweenness coefficient measures the role of the node $n_{i}$ as a bridge between nodes, a normalized closeness coefficient (the coefficient above multiplied by the number of nodes *n* − 1) may be seen as the inverse of the average shortest path distance between the node ni and all the nodes connected to $n_{i}$.

At network level, the most important measures are:

- **Degree distribution**: The degree distribution *p*(*k*) of a network is the fraction of nodes in the network with degree k. In a power law distribution, the fraction of nodes with degree k is *p*(*k*) ∝ *k* − *α* where *α* is a constant exponent. Networks characterized by such degree distribution are called scale-free networks. Many real networks such as the Internet topology [8], the Web [9] and on-line social networks [10] are often scale free.
- **Average path length**: In small-world networks, any two nodes in the network are likely to be connected through a short sequence of intermediate nodes, and the network diameter shrinks as the network grows [19].
- **Average clustering coefficient of a network**: The average clustering coefficient of a social network shows to what extent friends of a person are also friends with each other [33].
- **Density**: The density *D* of a network is defined as a ratio of the number of edges *E* to the number of possible edges, given by
  (3) $$D=\frac{2E}{N\left(N-1\right)}$$
  where N is the number of nodes in undirected graph, and $D=\frac{E}{N\left(N-1\right)}$ in directed graphs.
- **Small-world-ness coefficient**: The concept of “small-world-ness” as a property is exposed in [33], and characterizes networks with a high clustering coefficient (meaning by “high” as much higher than its equivalent in a random Erdos-Renyi network) and mean shortest path length similar to its equivalent in a random Erdos-Renyi network. Email users tend to form groups and the average shortest distance is small, leading to the small-world property.

In [34], a coefficient for measuring the small-world-ness of a network is presented and used as reference in this work. This small-world-ness coefficient is expressed as the ratio between *γg* and *λg*, where *γg* is the ratio between the average clustering network coefficient and the clustering coefficient of the network under the equivalent random Erdos-Renyi network, and *λg* is the ratio between the average shortest path length of the network and the average shortest path length of the equivalent random Erdos-Renyi network. 

The behavior of email networks have been generally claimed to be scale free, that is, the degree distribution follows a power law distribution. See for example [12,19,35]. The scale free nature of email data implies that a few ranges of nodes have high degrees (many friends) while many nodes have small degrees. 

In order to assess the scale free model for the degree distribution, log-log graphs or estimation of the power constant followed by a goodness of fit test are used. In this article, the goodness of fit method, through the use of a Kolmogorov Smirnov statistic, is applied. 

There are several email network studies in the literature. In [35], scale-free and small-world properties of an email network at Kiel University are studied. The Enron email database structure is studied in [18]. In [17], an email network from National Taiwan University is analyzed to study the temporal evolution of the email network of an EU research institution. In [19], we also see a focus on the temporal evolution of a large US University email network. 

The email database used in this study is a group of 29 independent email networks from Madrid Complutense University. This is the same as used in [32]. Each network is related to a University Faculty, and contains anonymized emails retrieved over one year between users of the department. Institutional emails, and emails going out of the Faculty network or coming from outside from the Faculty are not considered. Only time of sending and senders’ and receivers’ anonymized ID are kept. The textual content of email and headers are deleted. 

Studies on email network data may restrict the problem to a closed domain (considering only messages sent and received within a domain) or be open in the sense that messages outgoing from the domain and received from outside are also considered.

Most studies (see for example [18,19,35,36]) belong to the first category; others also consider out-domain emails ([19]). Most studies come from university or research institution servers.

Our study follows the norm; each network is restricted to the closed domain and data comes from a University server. The contribution here is that there are many separate similar networks (one per faculty), allowing for studying patterns and contrast previous research on this kind of data from a broader statistical view.

For simplicity and in order to avoid ambiguity, graph construction follows [16,19], creating an undirected and unweighted graph.

Table 1 presents the main measures of the 29 faculty networks. The smallest network has only 8 nodes and 23 edges, and the biggest has 622 nodes and 8839 edges. The average degree goes from 2.88 to 14.5. A 96% of the networks have an average degree > 4, in concordance with previous research on small-world networks, that reveals that the average degree is higher than 4. The average betweenness is in the range of (4.71, 2865). 

**Figure 1:**
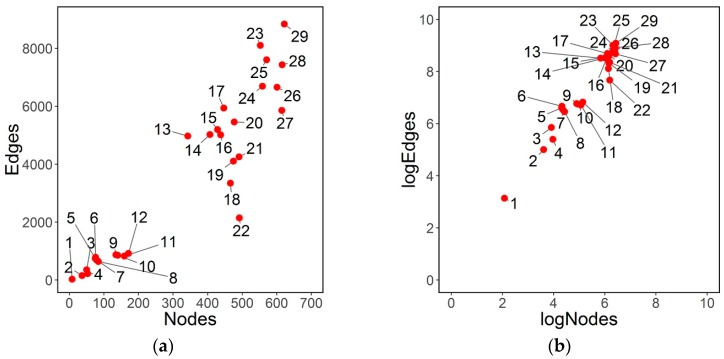
(**a**) Relationship between edges and nodes; (**b**) Relationship between edges and nodes in logarithmic scale.

It is shown in [34,37] that as density increases clustering coefficient increases and mean shortest path length approaches the equivalent random Erdos-Renyi network mean shortest path length. Thus high density networks would be trivially small-world under the WS concept. It is advisable that small-world behavior and coefficients should take into consideration the density concept for comparisons between networks. As [37] remarks, density should be lower than 0.4 in order to consider small-world-ness properties without the confounding effect of high density. Here the density range is very low, 80% of the networks show a density coefficient lower than 0.10, laying in the range of the other email networks studied in the literature. In Figure 1, the relationship between nodes and edges is represented. When transforming to log scale the relation is approximately linear, almost proportional, showing that

(4) log(edges)∝k×log(nodes)

That is $edges=nodes^{k}$. With the data presented here, *k* can be estimated by regression without intercept, giving *k* = 11.14, with regression *R*^2^ = 0.85.

Scale free behavior of the networks is evident. A Kolmogorov Smirnov test using bootstrap data presented in [38], is applied in order to check if the degree distribution in each network follows a power distribution. The null hypotheses of power distribution is only rejected for networks 11, 15, 22 and 27. The other 25 networks fit well to a power distribution, that is, $P\left(k\right)=k^{-\propto }$.

**Figure 2:**
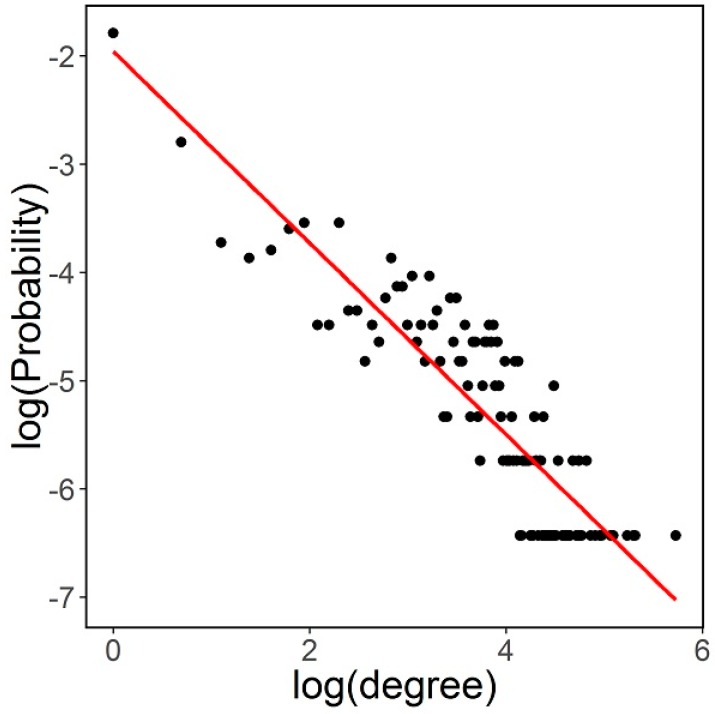
Log-log plot for Faculty 9 degree distribution.

The estimation of ∝ is achieved through maximum likelihood, following [38]. The estimate range is (1.43, 2.1). These values are similar to those found in [19], where data is limited to internal nodes of the closed network. Figure 2 and Table 2 show the degree distribution in log scale for faculty 6, and how it fits to a line.

### Small-World Behavior

Small-world networks are characterized by a high cluster coefficient Cg with respect to the equivalent random Erdos-Renyi network coefficient Cr, and similar path length Lg to the equivalent random Erdos-Renyi network Lr. Gamma and lambda values measure the quotients between each pair of coefficients. If the email networks considered here are small-world networks, it is to expect lambda values near 1 and high gamma values. In [34] a small-world-ness coefficient is presented constructed as the quotient between gamma and lambda values. A network can be considered small-world if this coefficient is higher than 2, what happens in 27 of the 29 faculties.

**Figure 3:**
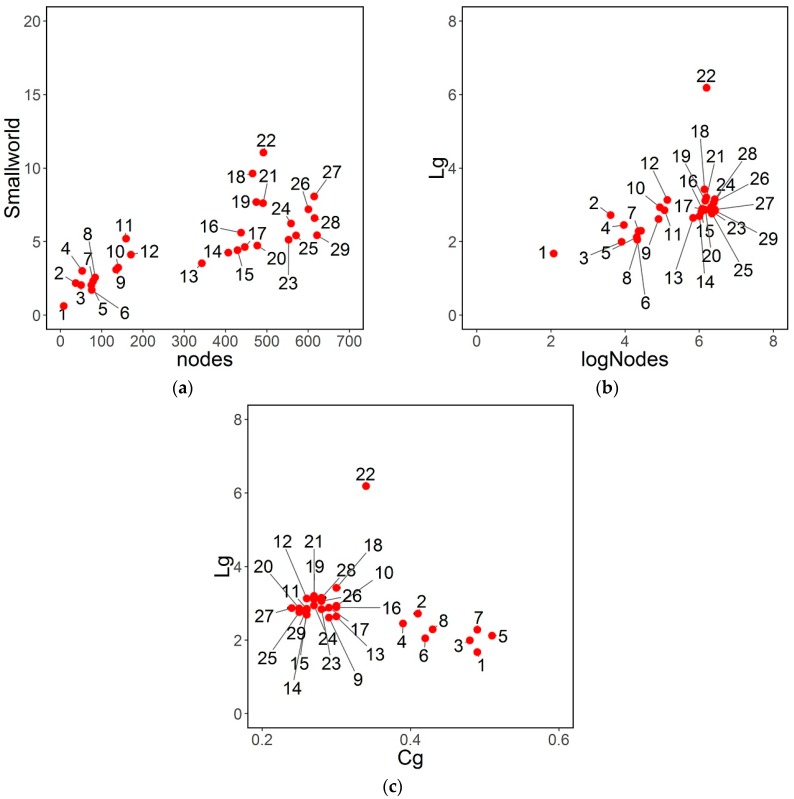
(**a**) Relationship between small-world-ness coefficient and number of nodes; (**b**) Relationship between shortest path and number of nodes; (**c**) Relationship between Lg and Cg.

In [34] it is pointed out that there is a linear relationship between small-world-ness coefficient and number of nodes. Figure 3a illustrates this fact. Also, it can be seen in Table 3 and Figure 3a that the faculties with smallest small-world-ness coefficient are those with less nodes, and it suggests that their small coefficient is due overall to their size and not to their structure in terms of shortest path and clustering.

Figure 3b allows also to detect in this case the network 22, which has an unusual behavior (higher Lg than expected). This network is also the one with highest shortest path, clustering coefficient, and small-world-ness coefficient. Figure 3c shows that mean shortest path and clustering coefficient have a special relationship. This seems to be increasing until a certain clustering coefficient and then decreasing when number of nodes is small and clustering coefficient is higher. It is possible that the clustering coefficient Cg has a special behavior when the number of nodes is too small. 

The regression slope (with case 22 deleted) for Cg < 0.35 is positive, *b* = 1.55 while the regression slope for Cg > 0.35 is negative, *b* = −4.29. Previous results on this relationship have not been found in literature. Faculty 22 is a special case (outlier), as it has been clear in other Figures and tables.

Other characteristics are proportional to the number of nodes. Figure 3b shows that

(5) Lg∝k×log(nodes)

## 3. Estimation of Email Network Characteristics through Statistical Disclosure Attacks

Privacy in communication networks can be compromised by statistical disclosure attacks. In this section it is shown how the method developed in [31,32] can be used to disclose user relationships (that is, existing and non-existing edges) in the network structure. Departing from very limited information, edges are inferred and users’ centrality measures and network global measures are estimated. This allows to detect high centrality nodes and characteristics of the network and establish the basis for studying network evolution with respect to global measures such as density, average degree, or average betweenness, and also node-based measures, when the attack is repeated at different time points.

The framework is habitual in statistical disclosure attacks in network communications: The information retrieved by the attacker is the number of messages sent and received by each user. This information is obtained in rounds that can be determined by equal length intervals of time, or alternatively by equal-sized batches of messages. Method is restricted, at this moment, to simple mix, where messages are grouped in batches at each round and then anonymously relayed, but can easily be extended to random threshold, where the batch size can be random, or pool mixes, where some messages are randomly selected and not relayed in each round. No restriction is made from before about the number of friends any user has, or about the distribution of messages sent. Both are considered unknown.

Attacker controls all users in the system. In our real data application, we aim at all email users of each network domain.

In each round, the attacker obtains a contingency table that represents messages sent from each user (rows) to each receiver (columns). Marginal row and column totals are known, and they represent the total number of messages sent and received by each user. However, the attacker does not know the pair (sender-receiver) for each message.

Table 4 represents a simplified version of one of these tables, retrieved in one round. There are many solutions for filling the table elements, that sum up to the marginals. Optimization algorithms (branch and bound) are generally slow and result in a very limited range of solutions. A very fast algorithm based on iterative random generation is used in [31] in order to obtain a large number of solutions (if not all) for each round. This information is used to order pairs of users from highest to lowest probability of relationship and finally obtain a classification result that aims to detect if one pair of users have had communication. In [32], a refinement of the method based on the use of the EM (Expectation-Maximization) algorithm that significantly improves the relationship predictions, is used.

In the network paradigm, the objective is to reconstruct the global network in the horizon of study, where nodes represent users and edges represent existing communication between pairs of users. The information employed to estimate the whole network is the incomplete information obtained in each round that can be seen itself as corresponding to a partial network with incomplete information.

The method leads to a final estimated network with its own measures and characteristics, that can be used as estimates of the real network measures. Besides, each user’s centrality measures can also be estimated.

As it was explained in [31,32], the performance of the attack is affected by the following aspects:

(1) The number of nodes. As the number of nodes increases, the complexity of round tables and the number of feasible tables increases, so that it negatively affects the performance of the attack.
(2) The percentage of existing edges over possible edges. As communication increases (more edges), the attack precision decreases.
(3) The mean frequency of messages per round (sum of weights in the round associated weighted network): This is directly related to the batch size, and when it increases, the performance is negatively affected.
(4) The number of rounds: As the number of rounds increases, this improves the performance of the attack, since more information is available.
(5) The number of feasible tables generated by round: This affects computing time, and it is necessary to study to what extent it is useful to obtain too many tables. This number can be variable. Usually once a high number of tables is generated (about 300,000 tables per round in our proofs), there is no gain in generating more tables.

The problem of estimating network characteristics departing from network incomplete data is often addressed in scientific literature under the subtitle ‘link prediction’. Personal information about individuals is generally used to predict relationships. There are seldom studies where only structural information is used. An exception is [13], where network characteristics are estimated in networks where only some nodes and links are known. It is a different context than the one addressed here, where all links are unknown and information is obtained in a multiple round framework.

The data presented in Section 2 allows us to study the precision and behavior of the attack when estimating users and network measures. Since there are 29 networks, homogeneous to some extent, estimators sensitivity to scale (number of edges) and other factors can also be observed.

For each of the faculty domains, construction of the attack follows the pattern below:

(1) Structure data in rounds. Messages are ordered by time and grouped by batch size B, forming rounds (each group of B messages is a round, that leads to a table similar to Table 4). In a real situation, this is the information the attacker is able to obtain.
(2) Develop the version of the attack algorithm presented in [32] and obtain an estimate of the adjacency matrix of the network; that is, an estimate of the whole network.
(3) Compute node centrality measures and network characteristics for the estimated network.

In order to develop this pattern a batch size needs to be decided on, as do the number of generated tables for each round, and number of iterations of the EM algorithm. Here a batch size of 15 is used, 500,000 tables per round are generated, and 5 EM iterations are developed.

**Figure 4:**
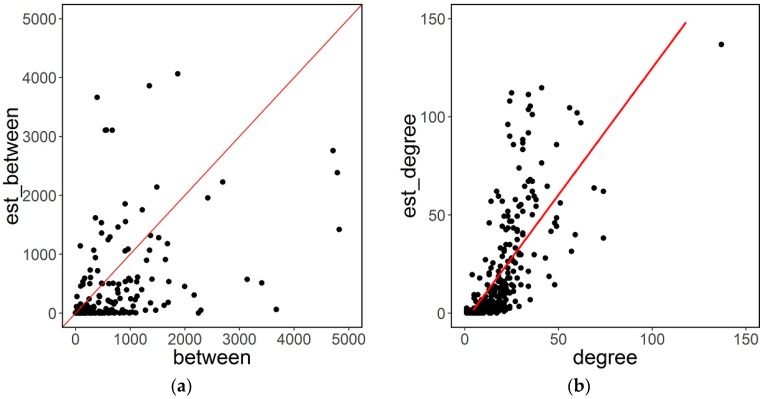
Relationship between estimates of (**a**) betweenness; (**b**) nodes degree, and its reciprocal real values for Faculty 16.

Centrality measures are computed for each node of each faculty network. Estimation error increases with size: error in degree or betweenness estimation is higher for nodes with higher degree or betweenness. Figure 4 represents the relationship between estimates of betweenness and node degree and their relative true values. In general, estimates are within the expected range. There is a slight but clear bias in both estimations: node degrees are slightly overestimated by the attack estimates, whereas betweenness is slightly underestimated. Uncertainty in round tables leads to the overestimation of edges (relationships) in the network and this has as a consequence higher degree values.

**Figure 5:**
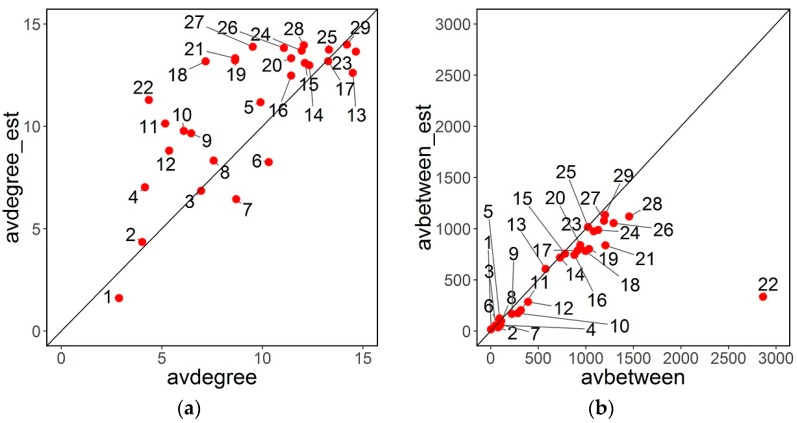

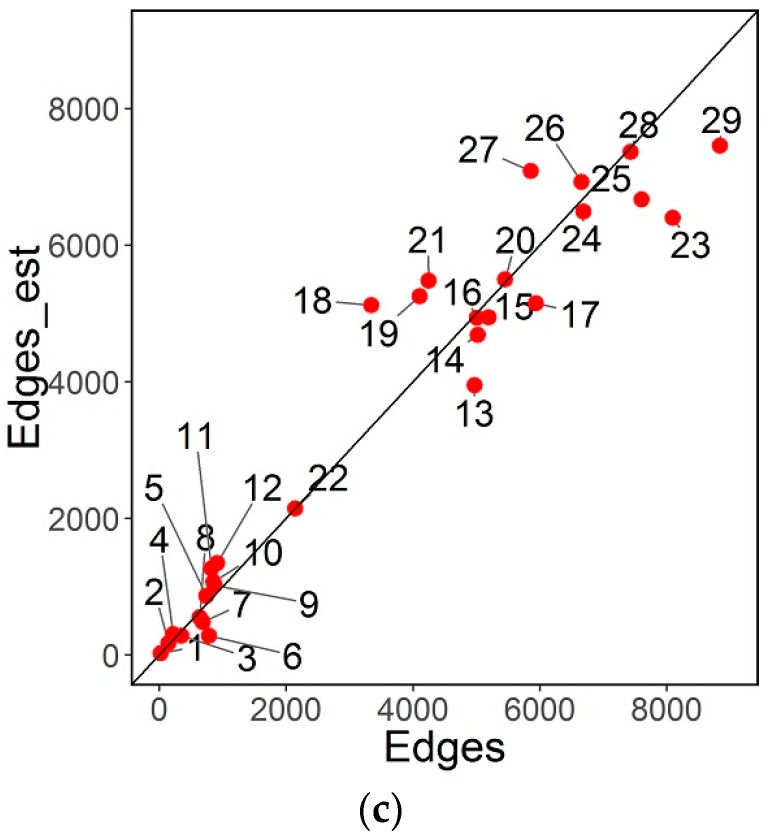
Estimation of (**a**) average degree (**b**) average betweenness (**c**) number of edges.

Figure 5 represents graphically the relationships between estimates and the true global values, for number of edges, average degree, and average betweenness. 

A line *y* = *x* is also represented to study possible biases. Some observations can be made:

- Estimation error, as it was expected, increases generally with network size (number of nodes).
- Number of edges for each network is slightly overestimated.
- Average degree is slightly overestimated, in concordance with user degree estimations.
- Average betweenness is slightly underestimated.

**Figure 6:**
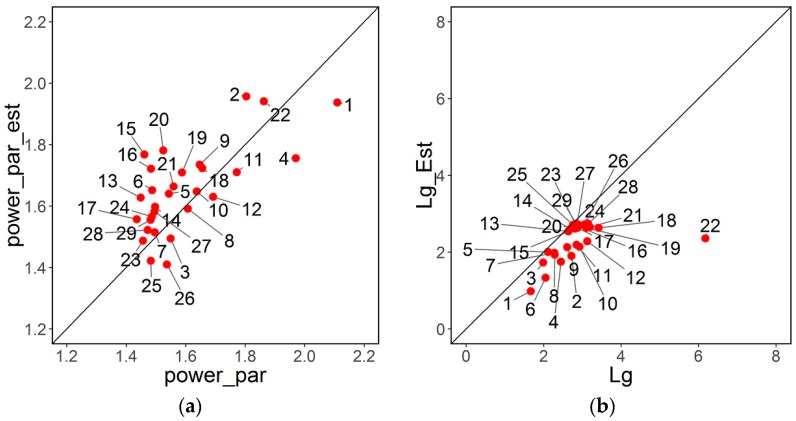

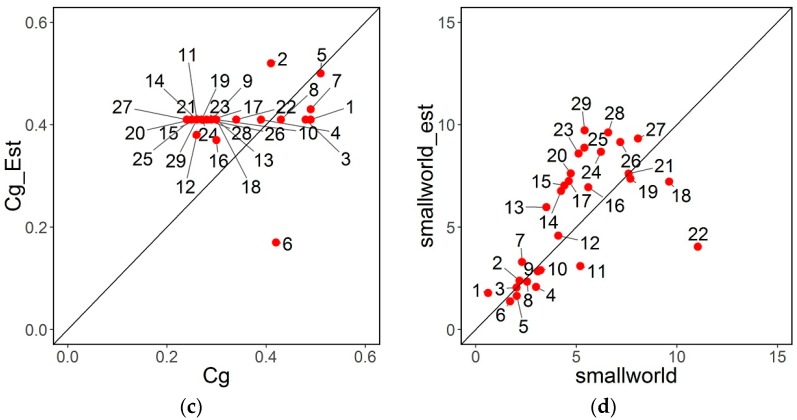
(**a**) Estimation of power distribution parameter; (**b**) Estimation of Lg; (**c**) Estimation of Cg; (**d**) Estimation of small-world-ness coefficient.

With respect to scale free behavior, the estimates of the power distribution parameter are slightly overestimated but within the true range, as is illustrated in Figure 6a.

Small-world characteristics are also estimated. Figure 6b–d present the relationship between the estimates and respective true values for Lg, Cg, and small-world coefficient. Mean shortest path Lg is slightly underestimated, whereas Cg and small-world-ness coefficient are slightly overestimated for large networks. With respect to the cutpoint of 2 for a small-world-ness coefficient, the estimator declares as small-world the same networks as the true value.

As it has been observed, estimation have often some bias. However, this bias is not so high and overall estimation error is accurate to a certain extent. It is known that the Mean Squared Error (MSE) of an estimator can be decomposed in two quantities:

(6) $$MSE=bias^{2}\left(est\right)+Var\left(est\right)$$

If both values are low in terms of the scale of the estimated quantity, the estimator is considered accurate. In Table 5 mean bias is computed for each network and parameter, as the mean of differences between the estimate and true value. Also, Mean Absolute Error (MAE) and CV are computed, CV representing MAE in percent over the mean value of the quantity estimated.

Estimation has much higher error at node level. At network level, estimators are satisfactory in general, within a controlled range of the quantity of interest. It is necessary to remark the limitations of the information used (all links are unknown a priori). At the network level, small-world-ness coefficient has the highest error in percent.

**Figure 7:**
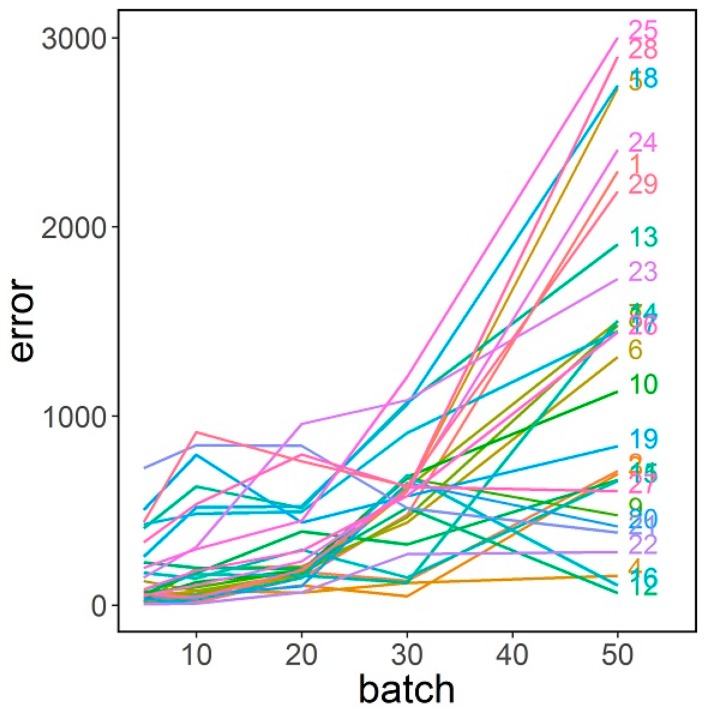
Absolute error in number of edges estimation versus batch size.

As it was pointed out, batch size significantly affects the performance of the attack. The attacker is limited to the number of times he can access information in batches. Figure 7 shows how the error in estimating the number of edges increases with batch size for all the faculties. As the number of edges is linearly or log-linearly related to most of the network measures, this has a direct consequence on the estimators’ errors.

## 4. Conclusions

Email networks are a particular case of interest in the field of Social Networks. Although these networks can share some characteristics with other social net systems, they have specific behaviors. In this work, a group of email networks with small size and low density are treated. It is usual in literature to mix network of different types in the same analysis, and sometimes the relationships between characteristics studied can be masked. With the data used in this article, homogeneity makes the findings about found relationships and characteristics more reliable. While most of them are already known it is interesting to observe them in a controlled context. Some observed findings are: Exponential relationship between edges and nodes, scale free behavior of almost all the email networks, small-world-ness behavior of almost all the email networks, linear relationship between small-world-ness and number of nodes, and linear relationship between shortest path and number of nodes.

Compromising the privacy of network communications is the aim of statistical disclosure attacks. Attacks can be developed to address information at multiple levels. In previous work, the aim of attack was to obtain diagnostics for relationships between each pair of users, detect communications, and classify each pair of users as linked or not. This work aims to obtain global characteristics of each node (centrality measures) that can be defined as a second level of privacy, and global characteristics of the network, as a third level of privacy. The utility of obtaining these measures can be exploited in two aspects: static information about special groups of nodes or comparing networks in different but homogeneous domains, and dynamic successive estimations over time. Characteristic measures of nodes and global network measures can be estimated at different points of time and will serve to study evolutions of nodes or networks.

Accuracy of the estimations is moderate, with some controlled bias in some measures. Estimators of the user characteristics have a higher error. In general, the range of estimations is similar to the objective range and results are considered encouraging due to the very limited information used. The attack is very affected by the batch size used; as batch size increases, accuracy decreases. 

The method used here in order to disclose and estimate network characteristics under very limited information may be improved if further information is available. If, for example, some links are already known, this information can be incorporated under the Bayesian paradigm to the basic algorithm results in order to refine estimations. The same approach could be used if one or more rounds are completely known in advance. This added information can also be used to apply bias correction to estimates that are slightly biased, such as average between and average degree estimates.

Even if there is no more information available, there is still space for improvement. There are other statistical disclosure methods such as the least squares method ([39]) that can be combined with the algorithm adopted here in order to refine results and correct biased estimates. Also, studying the network evolution over time may help to understand the estimation of network characteristics. Finally, the disclosure attack used here should be extended to more complex anonymous systems such as onion routing.

## Figures and Tables

**Table 1:** Faculty network centrality measures.

Faculty	Nodes	Edges	Avdegree	Avbetween	Density
1	8	23	2.88	4.71	0.41
2	37	149	4.03	80.36	0.11
3	50	348	6.96	51.92	0.14
4	53	221	4.17	100.00	0.08
5	75	743	9.91	94.47	0.13
6	76	784	10.32	80.76	0.14
7	79	687	8.70	101.06	0.11
8	84	637	7.58	109.71	0.09
9	135	872	6.46	223.64	0.05
10	140	852	6.09	286.93	0.04
12	171	918	5.37	394.37	0.03
13	343	4972	14.50	578.58	0.04
14	407	5024	12.34	728.92	0.03
15	429	5195	12.11	783.19	0.03
17	447	5938	13.28	916.15	0.03
11	159	822	5.17	318.34	0.03
16	438	5010	11.44	881.89	0.03
18	466	3345	7.18	998.50	0.02
19	475	4104	8.64	1034.46	0.02
20	477	5456	11.44	942.73	0.02
21	491	4249	8.65	1206.01	0.02
22	492	2143	4.36	2865.47	0.01
23	553	8100	14.65	1083.43	0.03
24	559	6688	11.96	1130.17	0.02
25	571	7606	13.32	1024.70	0.02
26	601	6657	11.08	1292.48	0.02
27	615	5858	9.53	1193.28	0.02
28	616	7435	12.07	1457.24	0.02
29	622	8839	14.21	1203.88	0.02

**Table 2:** Faculty scale free characteristics.

Faculty	Nodes	Edges	∝ Estimate	Power *p*-Value
1	8	23	2.1	0.85
2	37	149	1.8	0.56
3	50	348	1.54	0.96
4	53	221	1.97	0.39
5	75	743	1.54	0.54
6	76	784	1.48	0.78
7	79	687	1.49	0.63
8	84	637	1.60	0.22
9	135	872	1.64	0.09
10	140	852	1.63	0.12
11	159	822	1.77	0.01
12	171	918	1.69	0.26
13	343	4972	1.44	0.76
14	407	5024	1.49	0.16
15	429	5195	1.46	0.00
16	438	5010	1.48	0.37
17	447	5938	1.43	0.93
18	466	3345	1.65	0.37
19	475	4104	1.58	0.83
20	477	5456	1.52	0.21
21	491	4249	1.55	0.43
22	492	2143	1.86	0.00
23	553	8100	1.45	0.78
24	559	6688	1.48	0.73
25	571	7606	1.48	0.80
26	601	6657	1.53	0.27
27	615	5858	1.49	0.00
28	616	7435	1.47	0.14
29	622	8839	1.48	0.29

**Table 3:** Small-world characteristics. Faculties ordered by small-world coefficient.

Faculty	Lg	Lr	Cg	Cr	*λg*	*γg*	Nodes	Small-World
1	1.67	1.61	0.49	0.76	1.04	0.64	8	0.62
6	2.05	2.06	0.42	0.25	0.99	1.70	76	1.71
3	1.99	2.16	0.48	0.26	0.92	1.86	50	2.03
5	2.12	2.09	0.51	0.25	1.02	2.08	75	2.04
2	2.72	2.61	0.41	0.18	1.04	2.28	37	2.18
7	2.28	2.24	0.49	0.21	1.02	2.34	79	2.30
8	2.29	2.38	0.43	0.17	0.96	2.47	84	2.56
4	2.45	2.72	0.39	0.14	0.90	2.70	53	3.00
9	2.61	2.80	0.29	0.10	0.93	2.88	135	3.09
10	2.93	2.89	0.30	0.09	1.01	3.28	140	3.23
13	2.64	2.48	0.30	0.08	1.07	3.74	343	3.52
12	3.13	3.25	0.26	0.07	0.96	3.95	171	4.11
14	2.69	2.66	0.26	0.06	1.01	4.28	407	4.24
15	2.81	2.69	0.26	0.06	1.04	4.60	429	4.41
17	2.88	2.64	0.29	0.06	1.09	5.05	447	4.63
20	2.86	2.79	0.25	0.05	1.02	4.84	477	4.73
23	2.84	2.64	0.28	0.05	1.08	5.50	553	5.12
11	2.85	3.24	0.26	0.06	0.88	4.58	159	5.20
25	2.76	2.73	0.25	0.04	1.01	5.47	571	5.41
29	2.84	2.71	0.26	0.05	1.05	5.68	622	5.42
16	2.89	2.75	0.30	0.05	1.05	5.90	438	5.60
24	2.94	2.81	0.27	0.04	1.05	6.52	559	6.23
28	3.15	2.84	0.28	0.04	1.11	7.31	616	6.59
26	3.06	2.91	0.28	0.04	1.05	7.57	601	7.19
21	3.20	3.11	0.27	0.03	1.03	7.82	491	7.61
19	3.11	3.09	0.27	0.04	1.01	7.74	475	7.69
27	2.87	3.09	0.24	0.03	0.93	7.49	615	8.07
18	3.42	3.34	0.30	0.03	1.02	9.84	466	9.62
22	6.18	4.32	0.34	0.02	1.43	15.82	492	11.05

**Table 4:** Example of contingency table obtained in one round.

	U2	U4	U5	
U1				5
U2				4
U3				1
	3	5	2	10

**Table 5:** Mean Bias, Mean Absolute Error, and CV for main measures.

		Bias	MAE	CV
Node level	Degree	2.37	446	0.78
	Betweenness	−278	322	0.81
Network level: general	Edges	11.2	486	0.13
	Average degree	1.68	2.28	0.24
	Average between	−175	181	0.25
Network level: scale free	Degree distribution exponent	0.05	0.11	0.06
Network level: Small-world	Lg	−0.50	0.50	0.17
	Cg	0.076	0.11	0.33
	Small-world-ness coefficient	0.75	1.73	0.36
